# A developmental study of the effect of music training on timed movements

**DOI:** 10.3389/fnhum.2014.00801

**Published:** 2014-10-10

**Authors:** Thenille Braun Janzen, William F. Thompson, Ronald Ranvaud

**Affiliations:** ^1^Department of Psychology, Macquarie University, Sydney, NSWAustralia; ^2^Department of Neuroscience and Behavior, Institute of Psychology, University of São Paulo, São PauloBrazil

**Keywords:** rhythmic movements, timing, development, music training, discrete movements, continuous movements

## Abstract

When people clap to music, sing, play a musical instrument, or dance, they engage in temporal entrainment. We examined the effect of music training on the precision of temporal entrainment in 57 children aged 10–14 years (31 musicians, 26 non-musicians). Performance was examined for two tasks: self-paced finger tapping (discrete movements) and circle drawing (continuous movements). For each task, participants synchronized their movements with a steady pacing signal and then continued the movement at the same rate in the absence of the pacing signal. Analysis of movements during the continuation phase revealed that musicians were more accurate than non-musicians at finger tapping and, to a lesser extent, circle drawing. Performance on the finger-tapping task was positively associated with the number of years of formal music training, whereas performance on the circle-drawing task was positively associated with the age of participants. These results indicate that music training and maturation of the motor system reinforce distinct skills of timed movement.

## INTRODUCTION

Temporal entrainment refers to the rhythmic synchronization of movements to an external rhythmic signal, such as clapping along with the music (Phillips-Silver and Keller, 2012). Temporal entrainment also occurs when we coordinate our actions with other individuals, for instance, when people fall into simultaneous gait cadences (Nessler and Gilliland, 2009), play music in an ensemble (Maduell and Wing, 2007) or dance with another person (Brown et al., 2006; Bläsing et al., 2012; Phillips-Silver and Keller, 2012). Most people with no formal training can synchronize their movements to music. However, this apparently trivial phenomenon is dependent on a complex set of timing skills that are gradually developed through maturation of the motor system and can be greatly influenced by training. The present study examined the roles of music training and maturation in the development of timing mechanisms responsible for the precise production of discrete and continuous rhythmic movements.

Infants begin producing spontaneous movements in response to music from the age of 5 months (Eerola et al., 2006; Zentner and Eerola, 2010). There is no evidence, however, that children can produce rhythmic movements that are precisely timed to or synchronized with music before the age of 4 years (Drake et al., 2000; Provasi and Bobin-Bègue, 2003; McAuley et al., 2006; Zentner and Eerola, 2010; Morgan et al., 2013). Drake et al. (2000) noted that, although 4-year-old children can reproduce rhythmic patterns and synchronize to music, they are only able to do so within a restricted range of tempi. This range gradually expands between the ages of 4 and 10 years, suggesting that there are age-specific synchronization regions, and that the ability to process and produce discrete movements at different time spans improves gradually with age, reaching stable levels at adolescence (Drake et al., 2000; Baruch et al., 2004; Drewing et al., 2006; McAuley et al., 2006; Trainor and Corrigall, 2010).

In addition to the role of the natural development of the motor system in the production of rhythmic movements, studies have also suggested that various factors can influence the development of timing skills, such as social conditions, cultural context, and enriched experiences with music from a young age (Kirschner and Tomasello, 2009; Trehub and Hannon, 2009; Gerry et al., 2010; Hannon et al., 2011; Kirschner and Ilari, 2014). Not surprisingly, it has also been shown that formal music training significantly enhances precision of discrete movements (Drake et al., 2000; Baruch et al., 2004; Drewing et al., 2006; McAuley et al., 2006; Trehub and Hannon, 2009; Trainor and Corrigall, 2010; Hannon et al., 2011). For instance, Drake et al. (2000) demonstrated that children between 6 and 10 years old who received music training were significantly more accurate at rhythmic production tasks than age-matched counterparts. Drake (1993) showed that 7-year-old children who had music classes performed as well as non-musically trained adults in tasks that required the ability to reproduce rhythmic patterns. Research has also shown that adults with formal music training tend to be more accurate than non-musician counterparts in sensoriomotor synchronization tasks (Aschersleben, 2002; Repp and Doggett, 2007; Repp, 2010).

Most research on the development of the ability to coordinate rhythmic movements with music has focused on discrete movements, such as finger tapping, foot tapping, and clapping. However, not much is known about the effect of music training for continuous movements. Discrete rhythmic movements are defined as periodic actions that are preceded and followed by a phase without motion (Robertson et al., 1999; Huys et al., 2008; Studenka et al., 2012). Continuous rhythmic movements, on the other hand, are defined as smooth but periodic actions that lack clear action endpoints, and are typically assessed using tasks such as continuous circle drawing (Zelaznik et al., 2002; Huys et al., 2008; Zelaznik and Rosenbaum, 2010). Continuous circle drawing is often adopted as model to study emergent timing because it does not elicit salient perceptual events (e.g., visual, tactile, or kinesthetic), which could be used as reference to establish an explicit representation of the interval to be produced. In contrast, finger tapping has clear points in each cycle of movement when the action stops and then reverses, and involves contact with a surface, resulting in tactile, kinesthetic, visual, and auditory feedback that marks each movement cycle (Robertson et al., 1999; Zelaznik et al., 2002, 2005; Spencer et al., 2003; Zelaznik and Rosenbaum, 2010; Repp, 2011).

Besides the kinematic difference between discrete and continuous rhythmic movements, research suggests that different brain areas and cognitive processes are associated with discrete and continuous actions (Robertson et al., 1999; Zelaznik et al., 2002; Spencer et al., 2003; Schaal et al., 2004; Repp and Steinman, 2010; Studenka et al., 2012). In particular, discrete movements are based on event timing, involving a clock-like neural process and an explicit internal representation of the time interval delineated by each discrete movement. In contrast, activities that involve smooth and continuous rhythmic movements are thought to rely on emergent timing, whereby timing regularity emerges from the control of parameters such as movement velocity and trajectory control, and does not require an explicit representation of time (Robertson et al., 1999; Zelaznik et al., 2002; Huys et al., 2008; Studenka et al., 2012).

There is a need for more research on whether formal training in music influences the accuracy with which discrete and continuous movements are produced, and how music training might also affect the brain networks that are recruited to control these two types of movements. Studies have shown that music training significantly improves precision of discrete rhythmic movements (Drake et al., 2000; Aschersleben, 2002; Repp, 2005, 2010; Drewing et al., 2006) and recent research has indicated that music training improves precision in discrete but not continuous movements, suggesting that music performance relies primarily on timing mechanisms that require an explicit representation of time (event timing; Baer et al., 2013).

However, timed actions may also employ multiple mechanisms simultaneously. For example, playing the piano not only requires precise timing of the pianist’ keystrokes but also a fluid transition of the hand across the piano keys. Therefore, music performance may also require smoothly produced rhythmic movements. This suggestion supports the hypothesis that the distinction between event and emergent timing may not be as rigid as initially proposed, and that these mechanisms are not strictly tied to specific tasks but may both be adopted to achieve accurate timing (Jantzen et al., 2002, 2004; Repp and Steinman, 2010; Studenka et al., 2012; Studenka, 2014).

In the present study, we investigated whether formal music training enhances precision in discrete (finger tapping) and continuous movements (circle drawing). To this end, we examined the performance of children and adolescents from 10 to 14 years of age with a range of music training. We predicted that formal music training should reinforce event-timing strategies and hence have its largest effect on a discrete movement task (finger tapping). However, if musicians also perform more accurately than non-musicians on a circle-drawing task, then these results would suggest that music training also benefits the skills of continuous movements and their underlying mechanism. Such an outcome would raise the possibility that event timing and emergent timing are partially controlled by a common mechanism that is refined by music training, or that music training simultaneously enhances two independent timing mechanisms.

## MATERIALS AND METHODS

### PARTICIPANTS

Fifty-seven students (32 females, 25 males) were recruited from a private school in Sydney, NSW, Australia that offers music as a co-curricular activity. Eighteen students were recruited from Year 5 (13 females, 5 males) at primary school, and had an average age of 10.3 years (SD = 0.5). Of these, 10 had music training (*M* = 2.2 years; SD = 1.7) and eight had no music training (<2 years of training). Nineteen students were recruited from Year 7 (11 females, 8 males) and had an average age of 12.3 years (SD = 0.5). Of these, 11 were musicians (*M* = 4.1 years; SD = 2.1) and eight were non-musicians (<2 years of training). Finally, 20 students were recruited from Year 9 (9 females, 11 males) and had an average age of 14.3 years (SD = 0.5). Of these, 11 were musicians (*M* = 5.7 years; SD = 3.2) and nine were non-musicians (<2 years of training). All musically trained students were enrolled in music classes and were involved in at least 2 h of weekly musical activities; whereas non-musicians were not involved in any musical activity. All participants reported that they had no hearing or motor impairment. The Macquarie University Human Research Ethics Committee approved the experiment. Parents and caregivers were informed and debriefed about the goals of the experiment and gave consent for their child’s voluntary participation in this study.

### MATERIALS AND EQUIPMENT

Stimulus presentation and data collection were accomplished using MacBook *Pro* computers and custom software written in Python. The tones were produced by a Roland RD-250s digital piano and were presented over Sennheiser HD 515 headphones at approximately 74 dB SPL. Circle-drawing and finger-tapping tasks were completed with the right hand using the laptop mouse pad.

### STIMULI AND PROCEDURE

The continuation-tapping paradigm was adopted for both tasks (Stevens, 1886). For each trial, participants first synchronized their movements (circle drawing or finger tapping) with a series of 18 isochronous pacing signals. These pacing signals were a 480 Hz wave of 40 ms duration (square wave envelope), and intensity of 74 dB SPL as measured at the headphones. After the synchronization phase, the metronome stopped and participants continued to produce 36 more movements at the tempo set by the metronome. Within each trial, one of two metronome tempi was used: slow (800 ms interonset interval) or fast (600 ms interonset interval).

In the finger-tapping task, students tapped with their right index finger on the computer mouse pad at the tempo set by a pacing signal, and they continued to tap at the same rate when the signal was removed. In the continuation phase, every tap triggered a feedback tone, which was identical to the pacing signal. In the circle-drawing task, participants repeatedly traced a circle in a clockwise direction with their right index finger in time with the pacing signal. They continued this circular movement when the pacing signal was removed and replaced by a feedback tone. To assist with the circle-drawing task, an unfilled circle template of 5 cm in diameter was displayed on the computer screen. During the continuation phase, the feedback tone was triggered every time the path of the finger crossed the target point at 270^∘^ of the circle (9 o’clock). Participants were told that timing precision was more important than drawing accuracy, such they should feel free to draw the circle in any size. The mouse pad was configured at high sensitivity, and it did not produce any sound in response to finger tapping or circle drawing.

Participants had five trials to practice at 600 ms IOI before each testing block. Trials were blocked by task, and the order of presentation was counterbalanced between participants. Within each block, 16 trials were presented in random order: eight at the slow tempo and eight at the fast tempo. To control for outliers, trials in which inter-response intervals (IRIs) were 60% longer or shorter than the target IRI for a given trial were immediately discarded and re-done. With breaks offered between trials at the discretion of the participant, the task took approximately 30 min.

### DATA ANALYSIS

Only responses in the continuation phase were analyzed as the synchronization phase was used only to establish a consistent initial tempo of finger tapping or circle drawing. In order to avoid artifacts in the data arising from the acceleration of movement that is commonly observed in the transition between synchronization and continuation phases (Flach, 2005), only the final 31 cycles of movement were analyzed.

For the finger-tapping task, IRI was defined as the elapsed time between taps (in milliseconds). For the circle-drawing task, IRI was defined as elapsed time between passes of the index finger through the 270^∘^ intersection. To measure timing precision we analyzed each participant’s coefficient of variation (CV), which is defined as the standard deviation of IRIs within a trial divided by its mean IRI. CV can be considered a measure of total IRI variability, including slow drift in IRI over the course of a trial, timing error, and motor implementation error. The average CV was calculated across all trials for each condition and individual. Lower CV scores indicate greater timing precision. Dependencies between successive IRIs in each trial were also measured using lag-one autocorrelation. CV scores were averaged by task and tempo for each participant and entered into ANOVA with Task (circle drawing, finger tapping) and Tempo (fast, slow) as within-subjects factors, and Training (musicians, non-musicians) and Age (10, 12, 14) and as between-subjects factors.

## RESULTS

The analysis indicated a significant difference in timing precision (CV) between the two timing tasks, *F*(1,51) = 271.89, *p* < 0.005, suggesting that participants were more accurate in the finger-tapping task (*M* = 0.07) than in the circle-drawing task (*M* = 0.20). There was also a significant interaction between Task and Tempo, *F*(1,51) = 27.07, *p* < 0.005. More specifically, a repeated-measures *t*-test indicated that participants were more accurate on the circle-drawing task at a slow tempo (*M* = 0.19) than at a fast tempo (*M* = 0.23), *t*(57) = 7.02, *p* < 0.005, whereas timing accuracy for the finger-tapping task was similar for the slow (*M* = 0.07) and fast tempo conditions (*M* = 0.06, *p* = 0.40).

**FIGURE 1:**
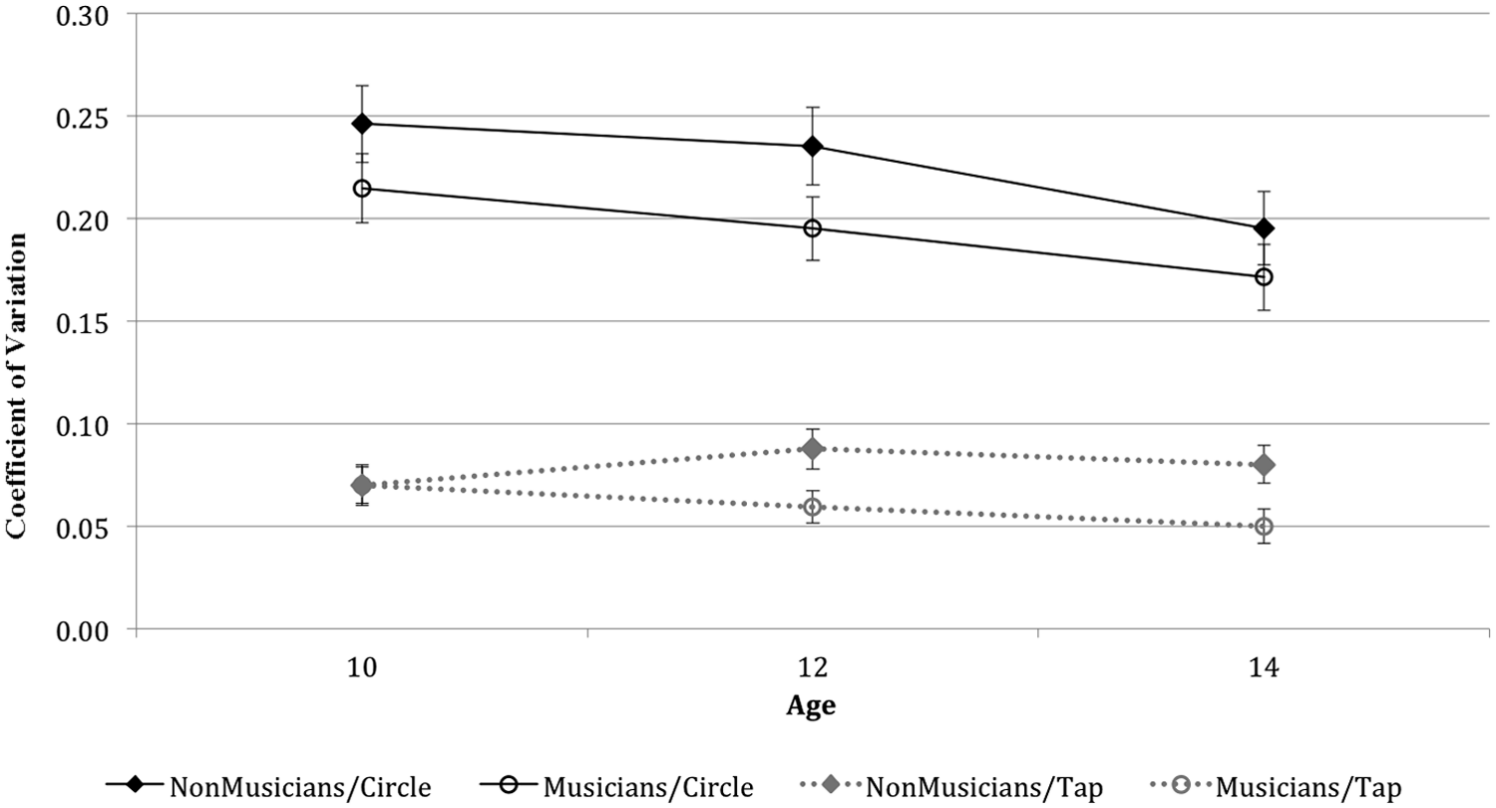
**Coefficient of Variation as a function of Task, Age and Group**.

Data analysis comparing the performance of musicians and non-musicians indicated a significant main effect of Music Training, with musicians performing significantly more precisely than non-musicians across the two timing tasks, *F*(1,51) = 6.59, *p* = 0.01. Although there was no significant interaction between Music Training and Task, pairwise comparisons suggested that the effect of music training is more reliable for the tapping task. The difference in timing precision for musicians and non-musicians was highly significant for the finger-tapping task (*p* = 0.006), but only marginally significant for the circle-drawing task (*p* = 0.06; see **Figure 1**).

The age of participants was also a significant factor in determining the precision in the performance of the timing tasks, *F*(2,51) = 3.38, *p* = 0.04. Pairwise comparisons showed that the participant’s age interacted with their performance on the circle-drawing task, in that 14-year-old students were significantly more precise than 10-year-old students (*p* = 0.02). However, age did not influence performance on the finger-tapping task (*p* = 0.26). To confirm this pattern of results, we conducted a correlation analysis with Task, Age, and Training. Results revealed a negative linear correlation between age and precision for the circle-drawing task (*r*^2^ = 0.29, *p* = 0.02), but not for the finger-tapping task (*r*^2^ = 0.21, *p* = 0.11). Conversely, Music Training was significantly correlated with performance on the finger-tapping task, (*r*^2^ = 0.41, *p* = 0.002), but not with performance on the circle-drawing task (*r*^2^ = 0.22, *p* = 0.08).

To further examine the effect of years of music training on the precision of discrete and continuous movements, we grouped musicians into three categories according to the average number of years of formal training (2, 4, and 6 years) and compared their performance with that of non-musicians. An independent-sample *t*-test analysis revealed that precision in the finger-tapping task was no different for musicians with 2 years of training (*M* = 0.07) than for non-musicians (*M* = 0.08), *t*(30) = 0.05, *p* = 0.95. On the other hand, musicians with an average of 4 years (*M* = 0.06) and 6 years of formal training (*M* = 0.05) performed significantly more precisely than non-musicians in the finger-tapping task (*p* = 0.03, and *p* = 0.004, respectively). There were no significant differences in the precision of circle drawing between non-musicians and any of the three groups of musicians.

Previous research has suggested that the introduction of salient feedback that demarcates each cycle of movement can induce event-timing strategies – even for continuous movements such as circle drawing (Zelaznik and Rosenbaum, 2010; Studenka et al., 2012). It has also been shown that musically trained and untrained individuals may adopt different strategies to maintain precise timing (Baer et al., 2013). To examine the timing strategies adopted by musicians and non-musicians to perform the finger-tapping and circle-drawing tasks, lag-one autocorrelation scores were analyzed (Wing and Kristofferson, 1973). Lag-one autocorrelation scores are predicted to be negative for event timing and non-negative for emergent timing (Wing and Kristofferson, 1973; Robertson et al., 1999; Zelaznik and Rosenbaum, 2010). Across groups, lag-one autocorrelation values were significantly less than zero for circle drawing (-0.05) and for finger tapping (-0.04, *p* < 0.005), suggesting that both tasks were performed using an event-timing strategy. There was no significant difference in the mean lag-one autocorrelation for musicians and non-musicians, either for the tapping task (*t* = 0.43, *p* = 0.66) or circle-drawing task (*t* = 1.46, *p* = 0.14). Moreover, lag-one autocorrelation values for the circle-drawing and finger-tapping tasks did not significantly differ (*p* = 0.57). Finally, there was a significant correlation between timing variability in the finger-tapping and circle-drawing tasks (*r*^2^ = 0.30, *p* = 0.02), further supporting the idea that participants adopted the same (event-timing) strategy to complete the two tasks.

## DISCUSSION

To better understand the role of music training on the development of timing skills involved in the control of discrete and continuous movements, we explored the question of whether maturation and formal music training interact with timing precision in finger-tapping and circle-drawing tasks. Our results indicated that musically trained students were significantly more accurate than non-musician counterparts in the finger-tapping task, and findings suggest that there was a significant correlation between years of formal training and precision of discrete rhythmic movements. We also found that musicians also tended to be more precise than non-musicians in the circle-drawing task, although the effect was not as strong as in the tapping task. The age of participants was significantly associated with performance on the circle-drawing task, suggesting that the motor control required for continuous movements is developmentally acquired and hence may become more stable after early adolescence.

Musically trained students performed significantly more accurately than non-musicians in the finger-tapping task, a finding that corroborates previous research implying that music training enhances precision of discrete movements (Drake, 1993; Drake et al., 2000; Aschersleben, 2002; Baruch et al., 2004; Drewing et al., 2006; McAuley et al., 2006; Repp and Doggett, 2007; Repp, 2010). The association between formal music training and the timing skills required to perform discrete rhythmic movements supports the hypothesis that musical activities refine the functioning of clock-like mechanisms that generate internal representations of the time interval delineated by discrete movements (Repp, 2005; Zelaznik et al., 2005; Baer et al., 2013). Through experience, practice, and years of formal training, event timing may be reinforced and emphasized. However, it should be acknowledged that an association between music training and timing precision could also arise because individuals with enhanced timing skills tend to gravitate toward music. Because our study adopted a quasi-experimental design, the nature of the association cannot be determined.

Nonetheless, the hypothesis that formal music training enhances timing skills involved in the control of discrete rhythmic movement is supported by a positive correlation between years of music training and precision of finger tapping. This finding suggests that practice in music interacts with timing mechanisms employed in discrete rhythmic tasks. This result is consistent with reports in the literature showing that the variability in sensoriomotor synchronization tasks of adult musicians can be as small as 2%, and 0.5% for percussionists (Repp, 2005), which indicates that years of extensive training lead to exceptional motor timing.

It was interesting to observe, however, that the effect of music training on the precision of discrete movements was only evident for students who had at least 4 years of music training. Musicians with an average of 2 years of training were not significantly more precise than non-musicians in the finger-tapping task. This finding suggests that, although music training can enhance timing skills, benefits may only emerge after extensive training. This suggestion is consistent with neuroimaging studies showing that structural changes in the brain are significantly associated with years of training in music (Gaser and Schlaug, 2003; Schlaug et al., 2005; Musacchia et al., 2008; Kraus and Chandrasekaran, 2010; Pantev and Herholz, 2011). In particular, Schlaug et al. (2005) noted that the impact of 4 years of music training on the plasticity of the brain is significantly stronger that the effect of 2 years of training to the child’s brain development.

Results also indicated that, although music training had its largest effect on the precision of discrete rhythmic movements (finger tapping), musicians were also more precise than non-musicians in the circle-drawing task, suggesting that music training may help to improve both types of timed movements. One interpretation of this pattern of findings is that timing mechanisms are not strictly tied to specific tasks but, as suggested by our lag-one autocorrelation analysis, the same (event) timing mechanism may have been adopted to achieve precise timing for both discrete and continuous timing tasks (Jantzen et al., 2002, 2004; Repp and Steinman, 2010; Studenka et al., 2012; Studenka, 2014). However, the number of years of music training was not significantly correlated with enhanced precision in the circle-drawing task, making it difficult to draw strong conclusions about the effects of music training on continuous-movement tasks. Future research is needed to evaluate the impact of extensive training in music and other movement-based activities (e.g., sports and dance) on continuous movements. Continuous rhythmic movements, such as leg movement during cycling, walking, and running, or arm movements during swimming or rowing, are typically observed in sport activities and dance (Sternad et al., 2000; Jaitner et al., 2001; Jantzen et al., 2008; Elliot et al., 2009). This class of rhythmic movements could be used as a model to study the effect of training in the production of precise continuous rhythmic movements.

Interestingly, our results showed that participants’ age was significantly associated with timing precision in the circle-drawing task, which suggests that the control of continuous movements is significantly associated with maturation of the motor system. These results indicate, therefore, that the ability to maintain accurate timing of continuous movements may be developmentally acquired. As such, such movements may not be highly stable in early adolescence but should improve with age. Studies investigating the development of the motor control required for the production of smooth hand movements in drawing and handwriting have also revealed significant age-related effects (Meulenbroek and Van Galen, 1988; Blank et al., 1999; Tseng and Chow, 2000; Robertson, 2001; Van Mier, 2006). Blank et al. (2000) showed that the different motor areas involved in the control of movements have distinct maturational periods. Moreover, Blank et al. (2000) demonstrated that the maturation of neuronal areas involved in the control of finger-generated actions is faster than the development of neuronal networks associated with arm and wrist movements. Van Mier (2006) studied the strategies children adopt to perform discrete and continuous movements, and noted that children of 4–5 years of age perform both movements in a discrete manner. These findings support the suggestion that timing strategies underlying continuous movements are not yet fully stable in early adolescence, whereas the neuronal network that control discrete movements are established earlier in life.

We also observed that the rate with which movements were made affected precision in the circle-drawing task but not in the finger-tapping task. For the circle-drawing task, participants were significantly more precise at the slow tempi than at the fast tempi. For the finger-tapping task, the precision of timing was unaffected by tempo. One interpretation of this finding is that the control of continuous movement at fast tempi may expose the limitations of a motor system still in development. Further research is needed to establish the most optimal rate within each developmental stage with which continuous movements can be timed precisely (see McAuley et al., 2006 for a review on age-specific entrainment regions of discrete movements).

Interestingly, we found that participants tended to adopt an event-timing strategy to perform both finger-tapping and circle-drawing tasks, as lag-one autocorrelation scores were negative for both tasks. The most probable explanation for this finding is the presence of an auditory feedback introduced at the completion of every movement cycle (Zelaznik and Rosenbaum, 2010; Studenka et al., 2012). Research has indicated that salient perceptual events (e.g., auditory, tactile feedback) significantly influence the timing strategy adopted to perform rhythmic movements, as the presence of auditory feedback generates an explicit internal representation of the temporal interval to be performed (Zelaznik and Rosenbaum, 2010; Studenka et al., 2012). However, Van Mier (2006) observed that younger children adopt event-timing strategies to perform both discrete and continuous tasks. Future studies are needed to understand whether education and training contribute to a differentiation between emergent and event-timing strategies, or whether the maturation of distinct neuronal networks predicts the use of different timing strategies to complete rhythmic tasks.

To conclude, this study showed that music training and the maturation of the motor system reinforce distinct skills of timed movement. Results showed that music training was associated with enhanced precision in the timing of discrete movements. Music training was also associated with the precision of timing for continuous movements, although to a lesser extent. The precision of continuous rhythmic movement was nonetheless associated with the age of participants, suggesting that the development of motor areas involved in discrete and continuous movement are subject to different maturation processes, and that the motor control required to produce continuous movements may develop more slowly than the motor control required to produce discrete movements.

## AUTHOR CONTRIBUTIONS

Thenille Braun Janzen was responsible for the experiment preparation, data collection and analysis, and manuscript preparation. William F. Thompson and Ronald Ranvaud provided feedback and suggestions regarding the writing of the manuscript.

## Conflict of Interest Statement

The authors declare that the research was conducted in the absence of any commercial or financial relationships that could be construed as a potential conflict of interest.
